# Clinical Utility of the 4S-AF Scheme in Predicting Atrial Fibrillation Recurrence after Radiofrequency Catheter Ablation

**DOI:** 10.31083/RCM26318

**Published:** 2025-03-14

**Authors:** Naiyuan Cui, Haiwei Li, Weiping Sun, Zefeng Wang, Zhongyu Yuan, Botao Zhu, Yutong Liu, Huanfu Liu, Yongquan Wu, Xiaoping Zhang

**Affiliations:** ^1^Department of Cardiology, Beijing Anzhen Hospital, Capital Medical University, National Clinical Research Center for Cardiovascular Diseases, 100029 Beijing, China; ^2^The Key Laboratory of Remodelling-Related Cardiovascular Diseases, Ministry of Education, Beijing Institute of Heart, Lung and Blood Vessel Diseases, 100029 Beijing, China

**Keywords:** atrial fibrillation, radiofrequency catheter ablation, recurrence, 4S-AF scheme, domain, atrial remodeling

## Abstract

**Background:**

The 4S-AF scheme, which comprises four domains related to atrial fibrillation (AF), stroke risk (St), symptom severity (Sy), severity of AF burden (Sb), and substrate (Su), represents a novel approach for structurally characterizing AF. This study aimed to assess the clinical utility of the scheme in predicting AF recurrence following radiofrequency catheter ablation (RFCA).

**Methods:**

We prospectively enrolled 345 consecutive patients with AF who underwent initial RFCA between January 2019 and December 2019. The 4S-AF scheme score was calculated and used to characterize AF. The primary outcome assessed was AF recurrence after RFCA, defined as any documented atrial tachyarrhythmia episode lasting at least 30 seconds.

**Results:**

In total, 345 patients (age 61 (interquartile range (IQR): 53–68) years, 34.2% female, 70.7% paroxysmal AF) were analyzed. The median duration of AF history was 12 (IQR: 3–36) months, and the median number of comorbidities was 2 (IQR: 1–3), and 157 (45.5%) patients had left atrial enlargement. During a median follow-up period of 28 (IQR: 13–37) months, AF recurrence occurred in 34.4% of patients. After eliminating the Sy and St domains, both the 4S-AF scheme (hazard ratio (HR) 1.38, 95% confidence interval (CI): 1.19–1.59, *p* < 0.001) and severity of burden and substrate of atrial fibrillation (2S-AF) scheme scores (HR 1.59, 95% CI: 1.33–1.89, *p* < 0.001) were independent predictors of AF recurrence following RFCA. For each domain, we found that the independent predictors were Sb (HR 1.84, 95% CI: 1.25–2.72, *p* = 0.002) and Su (HR 1.71, 95% CI: 1.36–2.14, *p* < 0.001). Furthermore, the 4S-AF (area under the curve (AUC) 65.2%, 95% CI: 59.3–71.1) and 2S-AF scheme score (AUC 66.2%, 95% CI: 60.2–72.1) had a modest ability to predict AF recurrence after RFCA.

**Conclusions:**

The novel 4S-AF scheme is feasible for evaluating and characterizing AF patients who undergo RFCA. A higher 4S-AF scheme score is independently associated with AF recurrence after RFCA. However, the ability of the 4S-AF scheme to discriminate between patients at high risk of recurrence was limited.

## 1. Introduction

Atrial fibrillation (AF) is a heterogeneous and complex cardiac arrhythmia with a steadily increasing global incidence and prevalence. AF can severely impair the quality of life of patients owing to its serious complications, including stroke, cognitive impairment, heart failure (HF), and death [1, 2, 3]. Radiofrequency catheter ablation (RFCA) can effectively preserve sinus rhythm and alleviate the burden of AF, thereby improving cardiovascular outcomes [4, 5, 6, 7]. RFCA is currently the first-line rhythm control therapy and cornerstone treatment for symptomatic, drug-resistant AF [5, 8].

However, AF recurrence rates following RFCA remain significant, with estimates ranging from 20% to 75% within two years of the initial procedure [9]. The recurrence of AF can be attributed to technical failure, extrapulmonary vein triggers, autonomic neural activity, and AF progression [8, 9, 10, 11]. Evidence from animal models and clinical studies indicates that atrial cardiomyopathy based on atrial fibrosis and atrial remodeling may be an underlying arrhythmia substrate that plays a pivotal role in AF progression and AF recurrence after RFCA [12, 13, 14]. In addition, major cardiovascular (CV) risk factors for AF, including aging, obesity, hypertension, diabetes mellitus (DM), HF, and coronary artery disease (CAD), promote atrial electrical and structural remodeling, leading to AF progression and AF recurrence [1, 15, 16, 17, 18, 19]. Therefore, there is an urgent need for simple tools and integrated models to assess the complex arrhythmia mechanism and predict AF recurrence after RFCA.

Several predictive models have been established to predict AF recurrence risk scores following catheter ablation, such as the BASE-AF2 (body mass index (BMI), left atrial diameter (LAD), smoking status, early recurrence, AF history duration and type), ALARMc (AF type, LAD, estimated glomerular filtration rate (eGFR), metabolic syndrome, cardiomyopathy)), and APPLE (age, AF type, eGFR, LAD, left ventricular ejection fraction (LVEF)) [20, 21, 22]. Recently, Potpara TS *et al*. [23] proposed a novel structured characterization outline for AF called the 4S-AF scheme, which encompasses four domains: stroke risk (St), symptom severity (Sy), severity of AF burden (Sb), and substrate (Su). Owing to its ability to facilitate AF assessment and therapy decisions, the 2020 European Society of Cardiology (ESC) AF guidelines have adopted this novel structured pathophysiology-based characterization scheme for comprehensive and personalized management [5]. Unlike the existing models, the 4S-AF scheme captures a more precise arrhythmia burden and a more comprehensive arrhythmia substrate evaluation. The 4S-AF scheme integrates broader aspects of the condition pathophysiology that existing models do not adequately address.

Emerging studies have shown that this novel scheme could provide prognostic information on AF progression and its adverse outcomes [24, 25, 26, 27, 28, 29]. Nonetheless, its clinical utility in patients with AF undergoing RFCA has yet to be established; therefore, this study aimed to determine the clinical utility of the 4S-AF scheme in predicting AF recurrence following RFCA.

## 2. Methods

### 2.1 Study Design and Population

This single-center, prospective, observational cohort study included a total of 545 consecutive patients with AF who underwent RFCA at Beijing Anzhen Hospital between January and December 2019. Inclusion criteria were: (1) age >18 years; (2) a confirmed diagnosis of AF and scheduled for RFCA treatment [5]; (3) voluntary agreement to participate in the study with signed informed consent. Patients were excluded if they had (1) long-standing persistent and permanent AF (n = 102); (2) previous major cardiac surgery history, including coronary artery bypass graft surgery (n = 7); (3) severe kidney dysfunction (eGFR <30 mL/min/1.73 m^2^) (n = 5); (4) previous RFCA history for AF (n = 29). Finally, the study cohort comprised 402 patients who all signed and provided informed consent. This study complied with the principles outlined in the Declaration of Helsinki for Human Research and received ethical approval from the Ethics Committee of Beijing Anzhen Hospital (Approval No: 2019198X).

### 2.2 Data Collection 

Baseline demographic, clinical, laboratory, and echocardiographic information were gathered from medical records. These data included patient age, sex, BMI, duration of AF history, AF classification, presence of comorbidities, and history of medication use. Two independent cardiologists calculated and determined the CHA_2_DS_2_–VASc score based on the provided guidelines [5]. Symptom severity was also categorized according to the European Heart Rhythm Association (EHRA) symptom score [5]. The HATCH score was also calculated using 1 point each for hypertension (H) and age ≥75 years (A), 2 points each for transient ischemic attack (TIA) or stroke history (T), 1 point for chronic obstructive pulmonary disease (C), and 2 points for HF (H) [30]. Echocardiographic parameters were measured before the operation using Philips 7C. Left atrial volume (LAV) was calculated using the prolate ellipse method with the formula: LAD1 (anterior - posterior) × LAD2 (superior - inferior) × LAD3 (medial - lateral) × 0.523 [31]. Body surface area (BSA) was determined using the Mosteller formula, while the left atrial volume index (LAVI) was calculated by dividing the LAV by the BSA [32].

### 2.3 4S-AF Scheme Characterization

Characterization based on the 4S-AF scheme was conducted for all patients and covered four domains: St, Sy, Sb, and Su (Table 1, Ref. [5, 23, 24, 25, 26, 27, 28, 29]). St was assessed if the stroke risk of the patient was higher than low and the patient had an indication for oral anticoagulant therapy based on the CHA_2_DS_2_–VASc score. Sy categorizations ranging from none to disabling were characterized using the EHRA symptom score. Sb was defined by the duration and frequency of AF episodes according to 2020 ESC guidelines for AF classification [5]. Su was characterized based on three subdomains: comorbidity/CV risk factors score, left atrial (LA) enlargement score, and age >75. Comorbidity was calculated based on the presence of the following conditions: hypertension, obesity (BMI >30 kg/m^2^), HF, hypercholesterolemia, DM, CAD, kidney dysfunction (eGFR <60 mL/min/1.73 m^2^), moderate or severe mitral valve regurgitation, and peripheral artery disease [23, 24, 25, 26, 27, 28, 29]. The total 4S-AF scheme score was derived by summing the values from each domain, yielding a maximum score of 10 (St = 1, Sy = 2, Sb = 2, and Su = 5). The severity of burden and substrate of atrial fibrillation (2S-AF) scheme score was adapted by excluding the Sy and St domains. The interpretation and definition for each domain are presented in Table 1.

**Table 1.** **Domains, interpretation, and definition of the 4S-AF scheme [23, 24, 25, 26, 27, 28, 29]**.

4S-AF scheme domains	Score	Interpretation	Definition
Stroke risk (St)			
	0	Low risk	CHA_2_DS_2_–VASc score^a^ 0 in males or ≤1 in females
	1	Not low risk, oral anticoagulation indicated	CHA_2_DS_2_–VASc score ≥1 in males or ≥2 in females
Symptoms (Sy)			
	0	No or mild symptoms	EHRA I and EHRA IIa
	1	Moderate symptoms	EHRA IIb
	2	Severe or disabling symptoms	EHRA III and EHRA IV
Severity of AF burden (Sb)			
	0	New or short and infrequent episodes	First diagnosed AF or paroxysmal AF
	1	Intermediate and/or frequent episodes	Persistent AF
	2	Long or very frequent episodes	Long-standing persistent AF or permanent AF
Substrate (Su)			
Comorbidity/CV risk factors			
	0	No	No comorbidity/CV risk factor
	1	Single	One comorbidity/CV risk factor
	2	Multiple	More than one comorbidity/risk factor
Left atrial enlargement			
	0	No	LAVI <29 mL/m^2^
	1	Mild–moderate	29 mL/m^2^ ≤ LAVI < 40 mL/m^2^
	2	Severe	LAVI ≥40 mL/m^2^
Age >75			
	0	No	≤75 years
	1	Yes	>75 years

AF, atrial fibrillation; EHRA, European Heart Rhythm Association; CV, cardiovascular; LAVI, left atrial volume index; 4S-AF, stroke risk, symptoms, severity of burden, and substrate of atrial fibrillation. ^a^The CHA_2_DS_2_–VASc score was calculated by summing the scores of C, clinical heart failure/left ventricular dysfunction/hypertrophic cardiomyopathy; H, hypertension; A2, age ≥75 years; D, diabetes mellitus/treatment with oral hypoglycemic drugs and/or insulin/fasting blood glucose >7 mmol/L; S2, stroke/thromboembolism/transient ischemic attack; V, previous myocardial infarction/peripheral artery disease/vascular disease/angiographically significant coronary artery disease/aortic plaque; A, age 65–74 years; Sc, female sex [5].

### 2.4 Radiofrequency Catheter Ablation Procedure 

After obtaining written informed consent, transesophageal echocardiography was performed to exclude atrial thrombus before undergoing RFCA. Procedures were carried out under local anesthesia. A mapping and ablation catheter was introduced into the left atrium via non-steerable long sheaths. The ablation process began with circumferential pulmonary vein isolation (CPVI), achieved by encircling the pulmonary vein ostia using irrigated ablation catheters (Thermocool SmartTouch©; Biosense Webster, Diamond Bar, CA, USA). The ablation procedure used a power control mode set to 35 W, with an irrigation flow rate of 17 mL/min. The ablation endpoint of CPVI was applied to establish a bidirectional conduction block between the left atrium and each pulmonary vein. Meanwhile, three-dimensional mapping was conducted using the PentaRay catheter (Biosense Webster, Diamond Bar, CA, USA). Additional atrial RFCA techniques were performed based on high-density voltage mapping to achieve AF termination if AF persisted after CPVI, such as cavotricuspid isthmus ablation, left atrium linear ablation, or low-voltage zone ablation. The procedural endpoint was the termination of AF. Intravenous heparin was continuously administered to maintain an activated clotting time between 300 and 350 seconds.

### 2.5 Outcome and Follow-up 

Follow-up assessments were performed by telephone, and regular visits were made to our outpatient clinics after ablation. Furthermore, patients were strongly recommended to obtain a 12-lead electrocardiogram (ECG) at the nearest hospital if they experienced any symptoms suggestive of arrhythmia or detected any irregular pulses through self-palpation or auscultation. The primary outcome was the recurrence of AF post-ablation, defined as any documented episode of atrial tachyarrhythmia (including AF, atrial flutter, or atrial tachycardia) lasting at least 30 seconds [11]. To accurately adjudicate outcomes, a detailed medical and physical examination, 12-lead ECG, and 24-hour Holter monitoring were performed at each visit and confirmed by trained cardiologists. Follow-up data were gathered from both telephone communications and outpatient medical records at Beijing Anzhen Hospital.

Regarding antiarrhythmic drug (AAD) management after RFCA, all patients received amiodarone or propafenone during the first 3 months after the procedure. This treatment was based on the 2020 ESC guidelines recommending AAD treatment for 6 to 12 weeks post-ablation to minimize early recurrence, rehospitalization, and cardioversion [5]. After 3 months, medication use was adjusted according to the individual clinical evaluation of each patient. Subsequently, AAD treatment was extended up to 6 months for those with AF recurrence.

### 2.6 Statistical Analysis

Analyses were performed using SPSS 24.0 (IBM Corp, Armonk, NY, USA) and GraphPad Prism 8.0.0 (GraphPad Software, San Diego, CA, USA). Data for continuous variables with a normal distribution are presented as the mean ± standard deviation (SD), while non-normally distributed variables are expressed as the median and interquartile range (IQR). Categorical variables are displayed as frequencies and percentages. Between-group comparisons for continuous variables were made using either the independent Student’s *t*-test or the Mann–Whitney U test, depending on data distribution. Categorical variables were analyzed using the chi-squared test or Fisher’s exact test, as appropriate. Cox regression analyses were conducted to identify risk factors for AF recurrence, incorporating each domain of the 4S-AF scheme as covariates. Multivariable Cox regression analysis was performed based on the 4S-AF and 2S-AF schemes after adjustment for age, gender, BMI, CAD, DM, HF, hypertension, stroke/TIA, atherosclerosis, and eGFR. Results are expressed as hazard ratios (HRs) with 95% confidence interval (CI). Kaplan–Meier survival curves were generated to evaluate time-dependent differences between groups, with the log-rank test used for comparisons. Receiver operating characteristic (ROC) curves and area under the curve (AUC) analyses were performed to investigate the predictive capability of the 4S-AF and 2S-AF schemes for AF recurrence. All statistical analyses were two-tailed, with a significance level set at α = 0.05. A *p*-value of <0.05 was considered statistically significant.

## 3. Results

### 3.1 Baseline Characteristics

**Fig. 1.**
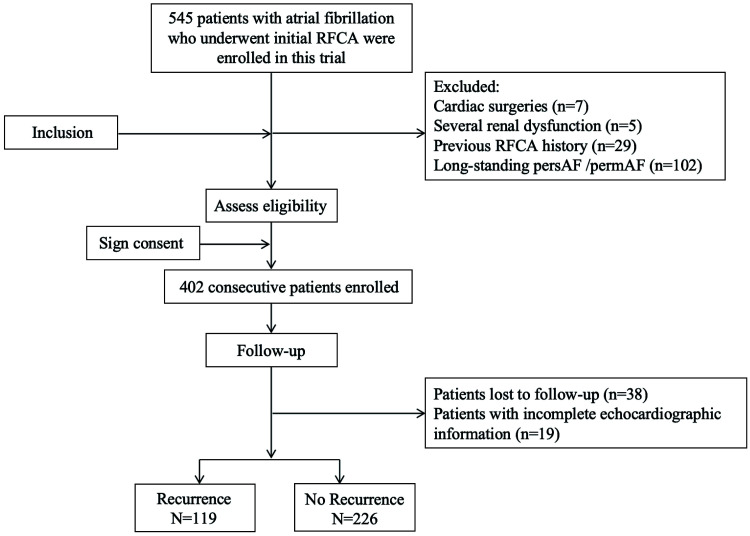
**Flowchart of the study**. RFCA, radiofrequency catheter ablation; persAF, persistent AF; permAF, permanent AF.

A total of 402 AF patients who underwent initial RFCA between January 2019 and December 2019 were consecutively included. Of these, 38 patients were lost to follow-up, 19 patients lacked complete echocardiographic data necessary for atrial substrate assessment; 345 patients were finally included in our analyses (Fig. 1). The median age was 61 (IQR: 53–68) years, 118 (34.2%) patients were female, and the median total duration of AF was 12 (IQR: 3–36) months. The most prevalent CV risk factors among the patients were hypertension (61.4%), HF (39.4%), CAD (16.8%), DM (19.1%), and obesity (16.5%). The recurrence group had a higher proportion of patients with HF, DM, and CAD than the non-recurrence group. Moreover, the CHA_2_DS_2_–VASc score was significantly higher in the recurrence group. Regarding the echocardiographic parameters, the LAD (42.0 (38.0–47.0) vs. 40.0 (38.0–43.2), *p* < 0.001), LAV (58.6 (46.8–72.0) vs. 50.8 (43.9–61.9), *p* < 0.001), and LAVI (30.8 (26.4–36.4) vs. 27.0 (23.5–32.3), *p* < 0.001) in the recurrence group were significantly greater compared to the non-recurrence group. Further clinical characteristics between the two groups are shown in Tables 2,3.

**Table 2.** **Baseline characteristics**.

Characteristics		Total (N = 345)	No recurrence (N = 226)	Recurrence (N = 119)	*p*-value
Age, years		61 (53–68)	61 (53–57)	63 (53–68)	0.311
Female, n (%)		118 (34.2)	73 (32.3)	45 (37.8)	0.305
Height, cm		170 (162–175)	171 (163–175)	168 (160–175)	0.079
Weight, kg		76.2 ± 12.9	76.6 ± 12.9	75.7 ± 13.3	0.567
Body mass index, kg/m^2^		26.7 ± 3.5	26.6 ± 3.4	26.9 ± 3.6	0.476
Systolic blood pressure, mmHg		129 (120–138)	129 (119–137)	128 (120–140)	0.855
Diastolic blood pressure, mmHg		80 (73–88)	80 (73–87)	80 (74–89)	0.466
Smoking history, n (%)		40 (11.6)	21 (9.3)	19 (16.0)	0.066
Drinking history, n (%)		46 (13.3)	28 (12.3)	18 (15.1)	0.477
Total history AF, months		12 (3–36)	12 (3–36)	12 (4–48)	0.432
Pacemaker history pre-ablation		13 (3.8)	9 (4.0)	4 (3.7)	1.000
AF classification, n (%)					0.023
	Paroxysmal	244 (70.7)	169 (74.8)	75 (63.0)	
	Persistent	101 (29.2)	57 (25.2)	44 (37.0)	
EHRA, n (%)					0.982
	I	18 (5.2)	11 (4.9)	7 (5.9)	
	IIa	185 (53.6)	123 (54.4)	62 (52.1)	
	IIb	135 (39.2)	86 (38.1)	49 (41.2)	
	III	7 (2.0)	6 (2.6)	1 (0.8)	
	IV	0 (0)	0 (0)	0 (0)	
Comorbidities, n (%)					
	Heart failure	136 (39.4)	79 (35.0)	57 (47.9)	0.019
	HFrEF	4 (1.2)	1 (0.4)	3 (2.5)	0.120
	HFpEF	132 (38.2)	78 (34.5)	54 (45.4)	0.048
	Hypertension	212 (61.4)	133 (58.8)	79 (66.4)	0.172
	Diabetes mellitus	66 (19.1)	36 (15.9)	30 (25.2)	0.037
	Coronary artery disease	58 (16.8)	29 (12.8)	29 (24.4)	0.006
	Stroke/TIA	30 (8.7)	21 (9.3)	9 (7.6)	0.588
	Atherosclerosis	82 (23.8)	47 (20.8)	35 (29.4)	0.074
	Obesity	57 (16.5)	32 (14.2)	25 (21.0)	0.103
	Hypercholesterolemia	28 (8.1)	21 (9.3)	7 (5.9)	0.270
CHA_2_DS_2_–VASc score					0.013
	0 or 1	130 (37.7)	97 (42.9)	33 (27.7)	
	2 or 3	129 (37.4)	75 (33.2)	54 (45.4)	
	≥4	86 (24.9)	54 (23.9)	32 (26.9)	
Medications, n (%)					
	Antiarrhythmic drugs	55 (15.9)	36 (15.9)	19 (16.0)	0.993
	β–blockers	133 (38.5)	89 (39.4)	44 (37.0)	0.663
	NDHP calcium channel blocker	2 (0.6)	2 (0.9)	0 (0)	0.547
	DHP calcium channel blocker	68 (19.7)	47 (20.8)	21 (17.7)	0.485
	ACE inhibitor	9 (2.6)	4 (1.8)	5 (4.2)	0.284
	Angiotensin receptor blocker	48 (13.9)	31 (13.7)	17 (14.3)	0.885
	Statins	44 (12.7)	31 (13.7)	13 (10.9)	0.460
	Diuretic	15 (4.3)	11 (4.9)	4 (3.7)	0.591
	Anticoagulant	147 (42.6)	92 (40.7)	55 (46.2)	0.325
	P_2_Y_12_ antagonist	8 (2.3)	5 (2.2)	3 (2.5)	1.000
	Aspirin	33 (9.6)	19 (8.4)	14 (11.8)	0.313
Echocardiographic parameters					
	LAD, mm	40.0 (38.0–44.0)	40.0 (38.0–43.2)	42.0 (38.0–47.0)	0.003
	LAV, mL	52.1 (44.9–64.3)	50.8 (43.9–61.9)	58.6 (46.8–72.0)	<0.001
	LAVI, mL/m^2^	27.9 (24.0–34.1)	27.0 (23.5–32.3)	30.8 (26.4–36.4)	<0.001
	LVEF, %	62.0 (59.0–66.0)	62.0 (59.0–66.0)	61.0 (58.0–66.0)	0.369
	LVM, g	158.8 (137.7–185.5)	158.8 (137.2–182.0)	158.8 (141.9–188.0)	0.539
	LVMI, g/m^2^	84.3 (74.3–97.2)	83.8 (74.1–96.0)	84.8 (75.3–100.2)	0.269

HFrEF, heart failure with reduced ejection fraction; HFpEF, heart failure with preserved ejection fraction; TIA, transient ischemic attack; NDHP, non-dihydropyridine; DHP, dihydropyridine; ACE, angiotensin-converting enzyme; LAD, left atrial diameter; LAV, left atrial volume; LAVI, left atrial volume index; LVEF, left ventricular ejection fraction; LVM, left ventricular mass; LVMI, left ventricular mass index.

**Table 3.** **Domains, definition, and characterization of the 4S-AF scheme**.

Domain		Total (N = 345)	No recurrence (N = 226)	Recurrence (N = 119)	*p*-value
Stroke risk (St) score, n (%)					0.002
	0: CHA_2_DS_2_–VASc score 0 in males or ≤1 in females	59 (17.1)	49 (21.7)	10 (8.4)	
	1: CHA_2_DS_2_–VASc score ≥1 in males or ≥2 in females	286 (82.9)	177 (78.3)	109 (91.6)	
Symptom severity (Sy) score, n (%)					0.924
	0: EHRA I–IIa	203 (58.8)	134 (59.3)	69 (58.0)	
	1: EHRA IIb	135 (39.2)	86 (38.1)	49 (41.2)	
	2: EHRA III–IV	7 (2.0)	6 (2.6)	1 (0.8)	
Severity of AF burden (Sb) score, n (%)					0.023
	0: First diagnosed AF or paroxysmal AF	244 (70.7)	169 (74.8)	75 (63.0)	
	1: Persistent AF	101 (29.3)	57 (25.2)	44 (37.0)	
	2: Long-standing persistent AF or permanent AF	0 (0)	0 (0)	0 (0)	
Substrate (Su) score, n (%)					<0.001
Comorbidity/CV risk factors score, n (%)					<0.001
	0: No comorbidity/CV risk factor	51 (14.8)	44 (19.5)	7 (5.9)	
	1: One comorbidity/CV risk factor	109 (31.6)	77 (34.1)	32 (26.9)	
	2: More than one comorbidity/risk factor	185 (53.6)	105 (46.4)	80 (67.2)	
Left atrial enlargement score, n (%)					<0.001
	0: LAVI <29 mL/m^2^	188 (54.5)	140 (61.9)	48 (40.3)	
	1: LAVI ≥29 mL/m^2^ and <40 mL/m^2^	126 (36.5)	75 (33.2)	51 (42.9)	
	2: LAVI ≥40 mL/m^2^	31 (9.0)	11(4.9)	20 (16.8)	
Age >75 score, n (%)					0.663
	0: ≤75 years	325 (94.2)	212 (93.8)	113 (95.0)	
	1: >75 years	20 (5.8)	14 (6.2)	6 (5.0)	
Each domain score, median (IQR)					
	St	1 (1–1)	1 (1–1)	1 (1–1)	0.002
	Sy	0 (0–1)	0 (0–1)	0 (0–1)	0.924
	Sb	0 (0–1)	0 (0–1)	0 (0–1)	0.023
	Su	2 (1–3)	2 (1–2)	2 (2–3)	<0.001
	4S-AF scheme score, median (IQR)	4 (3–5)	3 (2–4)	4 (3–5)	<0.001
Percentage of 4S-AF explained by each domain					
	St	25 (17–33)	25 (17–33)	25 (17–33)	0.651
	Sy	0 (0–25)	0 (0–25)	0 (0–20)	0.571
	Sb	0 (0–17)	0 (0–13)	0 (0–17)	0.066
	Su score	50 (50–67)	50 (40–67)	60 (50–67)	0.006
	2S-AF scheme score, median (IQR)	2 (1–3)	2 (1–3)	3 (2–4)	<0.001
Percentage of 2S-AF explained by each domain					
	Sb	0 (0–20)	0 (0–20)	0 (0–25)	0.074
	Su	100 (67–100)	100 (67–100)	100 (75–100)	0.498

CV, cardiovascular; IQR, interquartile range; 2S-AF, severity of burden and substrate of atrial fibrillation.

### 3.2 Characterization of 4S-AF Scheme

The characterization of patients according to the four domains in the 4S-AF scheme is detailed in Table 3. A majority of patients were identified as possessing a stroke risk other than low and required oral anticoagulant therapy (177 of 226 patients (78.3%) vs. 109 of 119 patients (91.6%), *p* = 0.002). The majority exhibited either no symptoms or mild symptoms based on the EHRA symptom score (EHRA I (5.2%), EHRA IIa (53.6%), EHRA IIb (39.2%), EHRA III (2.0%), and EHRA IV (0)). Additionally, most patients had more than one comorbidity, with the recurrence group exhibiting more comorbidities than the non-recurrence group. The median number of comorbidities was 2 (IQR: 1–3), indicating the presence of multiple coexisting conditions and/or CV risk factors in AF patients. Furthermore, over half of the patients in the recurrence group had mild to severe left atrial enlargement (86 of 226 patients (38.0%) vs. 71 of 119 patients (59.7%), *p* < 0.001), and more than half of those in the non-recurrence group had no left atrial enlargement (140 of 226 patients (61.9%) vs. 48 of 119 patients (40.3%), *p* < 0.001). The median score in the 4S-AF scheme was 4 (IQR: 3–5); for the 2S-AF scheme it was 2 (IQR: 1–3). According to our analysis, patients were significantly defined by the Su domain score, as shown in Table 3 (non-recurrence group, 50 (40–67); recurrence group, 60 (50–67), *p* = 0.006). However, the contributions by each domain to the 2S-AF scheme scores were comparable between the two groups.

### 3.3 Impact of 4S-AF Scheme on AF Recurrence Outcomes

**Fig. 2.**
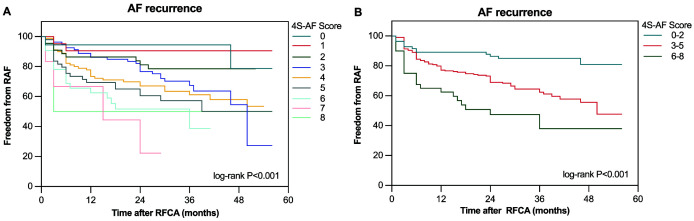
**The Kaplan–Meier curve demonstrated the difference in AF recurrence after RFCA between different 4S-AF scores**. (A) The Kaplan–Meier curve for AF recurrence after RFCA, based on 4S-AF scheme scores, is divided into nine groups (0–8 points). (B) The Kaplan–Meier curve for AF recurrence after RFCA based on 4S-AF scheme scores is divided into three categories (0–2 points, 3–5 points, 6–8 points). RAF, recurrence of atrial fibrillation.

**Fig. 3.**
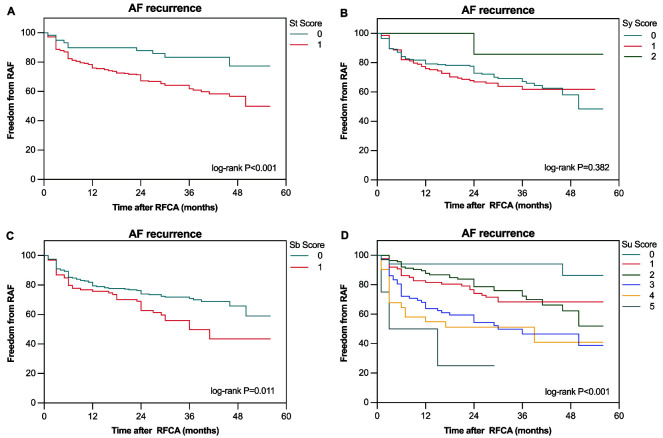
**The Kaplan–Meier curve demonstrated the difference in AF recurrence after RFCA between different scores for each domain: St domain (A), Sy domain (B), Sb domain (C), and Su domain (D)**.

The median follow-up period post-ablation was 28 (IQR: 13–37) months. Of the 345 patients analyzed, 119 (34.4%) experienced AF recurrence. The Kaplan–Meier survival curves indicated a significant association between higher 4S-AF scheme scores and increased AF recurrence rates (log-rank *p* < 0.001; Fig. 2). Moreover, the associations between each domain score and the risk of experiencing AF recurrence outcomes are shown in Fig. 3. Univariate Cox regression analysis established that the 4S-AF scheme score independently predicted AF recurrence (HR 1.37, 95% CI: 1.22–1.53, *p* < 0.001; Table 4). Meanwhile, after using the adjusted multivariable Cox proportional hazards models, we found that each one-point increment in the 4S-AF scheme score was significantly associated with a 38% higher risk of AF recurrence (adjusted hazard ratio (aHR) 1.38, 95% CI: 1.19–1.59, *p* < 0.001; Table 4).

**Table 4.** **Association of 4S-AF scheme score with AF recurrence after radiofrequency ablation**.

	Univariate model			Multivariate model		
	HR	95% CI	*p*-value	aHR^a^	95% CI	*p*-value
St	2.60	1.36–4.97	0.004	2.14	0.98–4.67	0.056
Sy	0.97	0.70–1.35	0.867	0.99	0.71–1.39	0.953
Sb	1.67	1.15–2.43	0.008	1.84	1.25–2.72	0.002
Su	1.59	1.35–1.86	<0.001	1.71	1.36–2.14	<0.001
4S-AF scheme score	1.37	1.22–1.53	<0.001	1.38	1.19–1.59	<0.001
2S-AF scheme score	1.53	1.33–1.76	<0.001	1.59	1.33–1.89	<0.001

HR, hazard ratio; aHR, adjusted hazard ratio. ^a^Adjusted for age, gender, diabetes mellitus, heart failure, hypertension, body mass index, coronary artery disease, atherosclerosis, estimated glomerular filtration rate, stroke/transient ischemic attack.

Analyzing the impact of each domain, the independent predictors of AF recurrence were identified as Sb (aHR 1.84, 95% CI: 1.25–2.72, *p* = 0.002) and Su (aHR 1.71, 95% CI: 1.36–2.14, *p* < 0.001) (Table 4). Moreover, the St and Sy domains did not significantly affect AF recurrence. Furthermore, the modified scheme score that combined the Sb and Su domain scores emerged as a stronger independent predictor of recurrence (aHR 1.59, 95% CI: 1.33–1.89, *p* < 0.001; Table 4).

### 3.4 Predictive Ability of 4S-AF Scheme in Patients Who Underwent RFCA

**Fig. 4.**
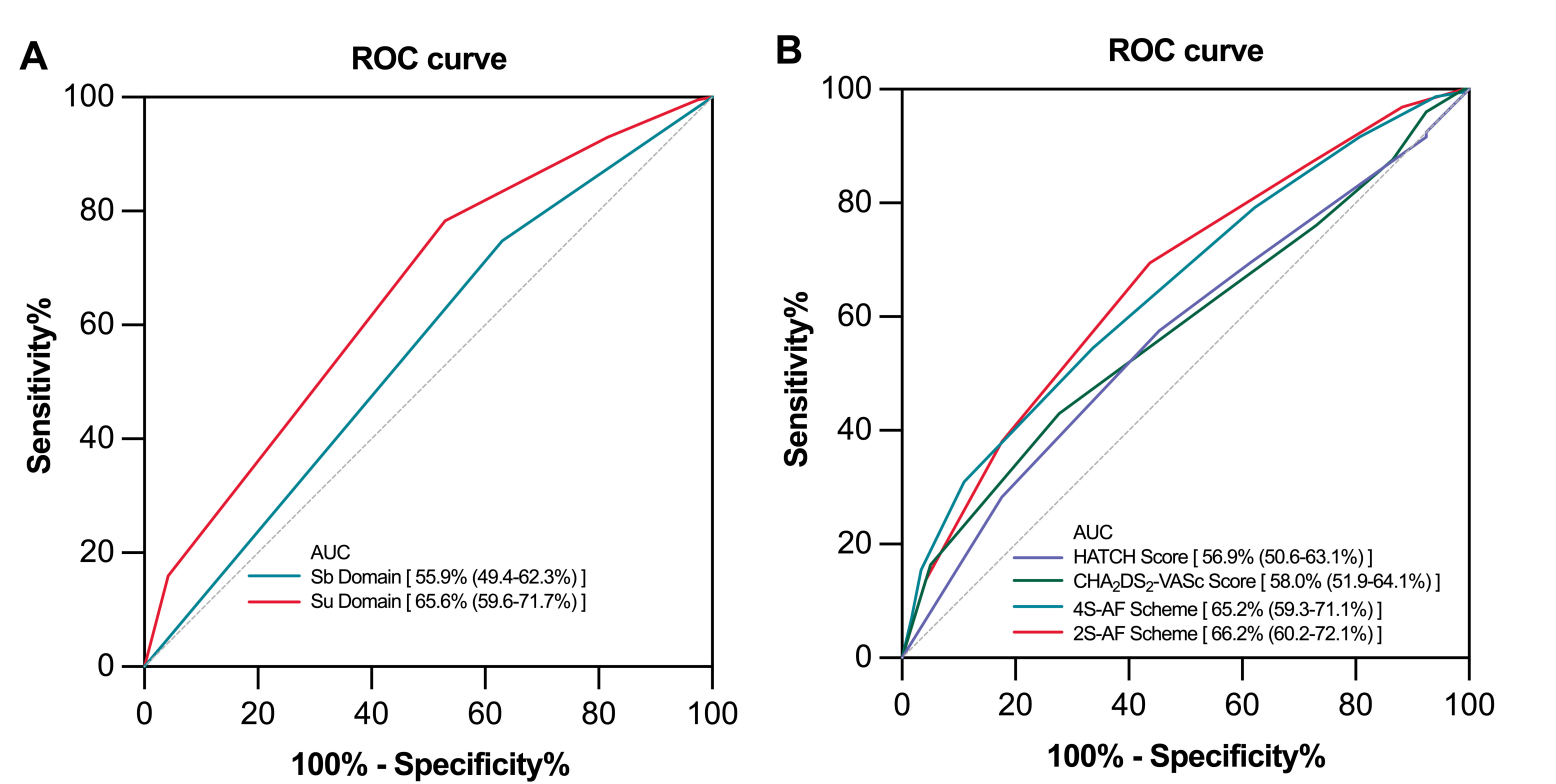
**Receiver operating characteristic (ROC) curves for predicting atrial fibrillation recurrence**. Receiver operating characteristic (ROC) curves for the Sb and Su domains (A); ROC curves for the 4S-AF scheme, 2S-AF scheme, CHA_2_DS_2_–VASc score, and HATCH score (B). AUC, area under the curve; HATCH, Hypertension-Age ≥75 years-Transient ischemic attack-Chronic obstructive pulmonary disease-Heart failure.

As shown in Fig. 4, our ROC analysis revealed that both the 4S-AF scheme score (AUC 65.2%, 95% CI: 59.3–71.1) and the 2S-AF scheme score (AUC 66.2%, 95% CI: 60.2–72.1) provided a modest predictive capability for AF recurrence. Compared to the HATCH (AUC 56.9%, 95% CI: 50.6–63.1) and CHA_2_DS_2_–VASc scores (AUC 58.0%, 95% CI: 51.9–64.1), the 4S-AF and 2S-AF scheme scores exhibited improved clinical predictive performances for AF recurrence. Subsequently, establishing a cutoff value of 3.5 for the 4S-AF scheme score further revealed that patients with scores ≥3.5 had a significantly higher risk of AF recurrence compared to those with scores <3.5 (HR, 2.121; 95% CI: 1.449–3.106, *p* < 0.001). Statistical analysis of the Z-score indicated no significant differences between the schemes (Z-statistic = –0.743, *p* = 0.458). Furthermore, the AUC values of the independent predictive ability for the Sb and Su domains were 55.9% (95% CI: 49.4–62.3%) and 65.6% (95% CI: 59.6–71.7%), respectively.

## 4. Discussion

### 4.1 Principal Findings

We prospectively investigated the clinical utilization of the 4S-AF scheme in predicting AF recurrence after RFCA. The principal findings were as follows: (1) Utilizing routinely collected data, the 4S-AF scheme is feasible for characterizing patients undergoing RFCA; (2) higher 4S-AF scores were significantly associated with increased AF recurrence rates, and the 2S-AF scheme, which integrates the Sb and Su domains, proved to be a stronger independent predictor of recurrence; (3) the Sb and Su domains scores were independently associated with AF recurrence outcomes after RFCA; (4) although the 4S-AF and 2S-AF schemes had limited predictive capability for AF recurrence after RFCA, they performed better than the traditional HATCH and CHA_2_DS_2_–VASc scores.

### 4.2 4S-AF Scheme and AF

The 4S-AF scheme represents a shift in AF classification towards a more structured and comprehensive approach. Unlike other AF classification systems, the 4S-AF scheme offers a practical assessment framework previously endorsed and recommended in the 2020 ESC guidelines for AF management [5]. EORP-AF Long-Term General Registry data analysis demonstrated that the novel scheme provided prognostic information on adverse outcomes related to AF, including thromboembolic events, CV, and all-cause mortality [29]. Notably, management decisions guided by this scheme have been linked to reduced all-cause mortality in AF patients [24, 33]; similar findings were observed in the APHRS-AF registry and FAMo cohort studies [27, 28]. An analysis of the RACE V data recently indicated that the modified 4S-AF scheme may predict AF progression in the subset of self-terminating AF patients after eliminating the Sy domain [25]. Furthermore, the 4S-AF scheme has prognostic implications for all-cause mortality and is a pragmatic risk stratification tool for patients with new-onset AF after myocardial infarction [26]. Nonetheless, studies assessing and validating the clinical utility and prognostic capability of the scheme in AF patients after RFCA remain limited.

Each domain in the 4S-AF scheme is highly practical for characterizing and evaluating AF because the scheme collects routine data on demography, comorbidities, AF-related symptoms, AF burden severity, and left atrial substrate. St is based on the CHA_2_DS_2_–VASc score, and the Su domain score comprises three subdomains, including the number of comorbid conditions/CV risk factors, atrial enlargement, and older ages. Hence, using this risk assessment tool, the St and Su domains partly depended on comorbidities when characterizing AF patients. However, the scheme is not without limitations, whereby the definitions of the comorbidities and CV risk factors need to be clarified, and some conditions might be neglected during routine clinical practice. For example, this study might have underestimated the risk of HF with preserved LVEF due to the limited use of invasive exercise hemodynamic assessments [34]. To evaluate atrial remodeling, we simply assessed left atrial enlargement using Doppler ultrasound, which may lead to the neglect of early atrial remodeling manifesting as atrial dysfunction [25, 35, 36]. Consequently, the total St and Su domain scores in the current analysis were lower than in previous studies. Additionally, the Sy domain score was higher in our cohort than those reported in the EORP-AF Long-Term General Registry and APHRS-AF Registry, likely because our population consisted of symptomatic AF patients scheduled for RFCA [28, 29]. Moreover, the Sb domain score was lower than that of other studies since we excluded long-standing persistent and permanent AF.

### 4.3 AF Recurrence after RFCA in the 4S-AF Scheme

To our knowledge, this study is the first to apply the 4S-AF scheme to characterize AF patients undergoing RFCA and assess utilizing the 4S-AF scheme in predicting AF recurrence risk post-ablation. Our analysis demonstrated that both higher 4S-AF and 2S-AF scheme scores were independently associated with an elevated risk of AF recurrence. This aligns with findings by Chollet L *et al*. [37], who confirmed that combining AF phenotype with LAVI offers prognostic value for AF recurrence following CPVI. Similarly, only the Sb and Su domains emerged as independent predictors of recurrence in our study after multivariable adjustment. Although the AUC values for each scheme fall below the threshold typically considered for strong discriminative power, it is important to recognize that these scores still offer valuable insights into the recurrence risk after RFCA. The clinical utility, prognostic value, and modified 4S-AF and 2S-AF schemes are highly desirable for predicting AF recurrence after RFCA and require further investigation. Numerous studies have emerged that have validated using several novel biomarkers, such as electrocardiographic, molecular, and imaging, to independently predict AF recurrence outcomes after RFCA [38, 39, 40, 41, 42, 43]. Combined with these novel biomarkers and other well-established scoring systems, the predictive ability and reclassification performance of the scheme may be significantly improved among patients who undergo RFCA [44]. Since identifying atrial remodeling and monitoring cardiac rhythm using routine tools in clinical practice is challenging, most descriptors of AF domains remain inadequately defined and have not been evaluated accurately. Thus, future studies should focus on refining the 4S-AF scheme, possibly by incorporating additional parameters such as atrial structural and functional markers and validating its prognostic utility in larger, more diverse populations. We believe the 4S-AF scheme has great clinical utility and prognostic value, with future refinements guided by advanced cardiac imaging and rhythm screening technology [45].

While the 4S-AF scheme was not initially designed for risk stratification after RFCA, our prospective cohort demonstrated its meaningful predictive value for AF recurrence. Moreover, applying the 4S-AF scheme in a post-RFCA setting helps identify patients with a higher AF recurrence risk, thereby enabling targeted monitoring and interventions to improve long-term outcomes. However, data from the EORP-AF Long-Term General Registry indicated that greater 4S-AF scheme scores were suitable for more aggressive interventions to improve the adverse long-term outcomes of AF [29]. Hence, using the 4S-AF scheme to predict different post-RFCA outcomes and the weighting of each domain need future validation to streamline the selection of AF patients who may benefit from RFCA.

### 4.4 Study Limitations

We acknowledge several limitations of the current study. First, we included only paroxysmal and persistent AF patients who underwent initial RFCA at our center in this analysis; thus, the exclusion of other AF patients may be a source of bias. Therefore, future studies should validate the conclusions in more diverse cohorts of AF patients after RFCA. Second, all patients in our study underwent a standardized approach to RFCA, beginning with CPVI and incorporating additional substrate modification and linear ablation techniques as needed. While the approach remained consistent, it is well-established that variations in ablation strategies, particularly in patients with persistent versus paroxysmal AF, can influence outcomes. Due to the observational nature and lack of detailed procedural data for this study, we could not assess how specific ablation techniques directly influenced recurrence rates. Moreover, the traditional tools available to determine AF episodes and burden may underestimate AF recurrence compared with implanted or continuous rhythm monitoring devices [25, 46]. The 4S-AF scheme is a dynamic score, but we only assessed it at baseline, and limited follow-up data was available for periodic reassessment. Furthermore, there is some overlap between the four domains. Finally, the study was performed at a single center with a relatively small sample size. AF is a complex condition; thus, we cannot exclude residual confounding [3].

## 5. Conclusions

We demonstrated that the 4S-AF scheme is practical and feasible for evaluating and characterizing AF patients who undergo RFCA. A higher 4S-AF scheme score is independently associated with AF recurrence after RFCA. However, the ability of the 4S-AF scheme to discriminate patients at high risk of recurrence among various AF characterization remains limited. These findings provide a foundation for future research to improve risk stratification for AF recurrence post-RFCA.

## Availability of Data and Materials

The data underlying this article will be shared on reasonable request to the corresponding author.
